# Impact of the Covid-19 pandemic on primary care utilization: evidence from Sweden using national register data

**DOI:** 10.1186/s13104-021-05839-7

**Published:** 2021-11-24

**Authors:** Björn Ekman, Eva Arvidsson, Hans Thulesius, Jens Wilkens, Olof Cronberg

**Affiliations:** 1 Department of Clinical Sciences, Malmö, Lund University, Jan Waldenströms gata 35, 202 05 Malmö, Sweden; 2 Futurum, Region Jönköping’s County, Jönköping, Sweden; 3 School of Health and Welfare, Jönköping University, Jönköping, Sweden

**Keywords:** Covid-19, Primary care, Service delivery, Sweden

## Abstract

**Objective:**

To analyze changes in primary care utilization as a result of the Covid-19 pandemic. Swedish national register data from 2019 to 2020 on utilization of services were used to compare overall utilization levels and across types of contacts and patient groups. A specific objective was to assess the extent to which remote types of patient consultations were able to compensate for any observed fall in on-site visits. Data were stratified by sex and age to investigate any demographic pattern.

**Results:**

Findings show significant reductions in overall utilization of services as the pandemic occurred in the first quarter of 2020. On-site visits fell during the first wave of the pandemic and rebounded thereafter. Patients over 65 years of age appear to have reduced utilization to a larger extent compared with younger groups. Simultaneously, remote contacts increased from around 12% before the pandemic to 17% of the total number of consultations. However, the net effect of changes in service utilization suggests an overall reduction of around 12 percent in the number of primary care consultations as a result of the pandemic. No differences between men and women were observed. Further research will continue to monitor changes in primary care utilization as the pandemic continues.

**Supplementary Information:**

The online version contains supplementary material available at 10.1186/s13104-021-05839-7.

## Introduction

As the Covid-19 pandemic began to spread across the world in the first quarter of 2020 the utilization of on-site health care services for non-covid related conditions fell in most countries and regions [1]. The reduction in clinic-based visits was the result of both supply and demand side effects. To reduce the spread of infections clinics were either mandated to limit on-site visits or encouraged to do so [2]. Likewise, many patients refrained from making clinic visits to decrease the risk of becoming infected [3, 4].

In parallel, the utilization of various types of remote contacts increased from current long-term trends [5–7]. The relatively rapid increase in telemedicine contacts during the initial phases of the pandemic can be seen as an indication of resilience on the part of the health system to maintain service levels in the face of an external shock. However, to fully understand the impact of the pandemic on the system’s ability to continue delivering services during the pandemic, analyses need to adopt a more comprehensive approach than has generally been the case to date. First, pre-pandemic utilization trends need to be described in order to obtain a relevant baseline measure. Second, data on all types of patient consultations need to be included in the analysis. And third, monitoring of health care utilization needs to be sustained over several waves of the pandemic to assess providers’ and patients’ behaviors over time. Using national, register-based data on both on-site visits and remote primary care contacts in Sweden for 2019 and 2020, this study describes the changes in primary care service utilization during the first year of the Covid-19 pandemic. The current study forms part of a larger research project that aims to analyze the impact of the Covid-19 pandemic on health care service provision in Sweden.

## Main text

### Methods

#### Study design

The study adopts a cross-sectional, time-series design using daily patient primary care utilization data for 2019 and 2020 from Sweden. The data source is the Väntetidsdatabasen (VTDB), which is a national database on all primary care consultations in the country. It includes consultations with physicians, nurses, physiotherapists, and other care professionals. Indicators include age, sex, diagnosis, and type of consultation. The database was initiated in 2019 and not all regions reported complete data in the first year. After adjusting for missing baseline values the total sample sizes are 12,079,268 (2019) and 10,847,918 (2020) primary care consultations, covering two-thirds of the 21 Swedish health care regions and around 4 million people.^1^ Primary care in Sweden is provided by public and private multi-professional clinics that are reimbursed by means of a combination of capitation and fee-for-service by the regional health authorities. Most adult patients pay a nominal user fee per primary care consultation (see, e.g., [8] for details on the Swedish health care system).

#### Measures

The total number of consultations per time unit (month) for each year is the main measure of the study. To analyze the relevant changes that took place as a result of the pandemic this indicator is disaggregated into a set of measures as follows. First, the total number of consultations per month for each year is described. Second, the total number of consultations is then reported separately for on-site visits (clinic and home visits) and remote contacts (video- and chat-based, and telephone and letter contacts). And third, the number of consultations is described across five separate age categories and by sex, respectively. In addition, the total number of confirmed Covid-19 cases and deaths are shown to provide the context of the study.

#### Analysis

All data analysis was conducted using Stata 16.1 (www.stata.com). The identified measures are reported as absolute numbers and as percentages when relevant. The results of the analysis are presented in separate graphs and the aggregate data are presented in tables. The main results are presented in the main text and additional findings are provided in the Additional file.

### Results

#### The Covid-19 pandemic in Sweden

**Fig. 1:**
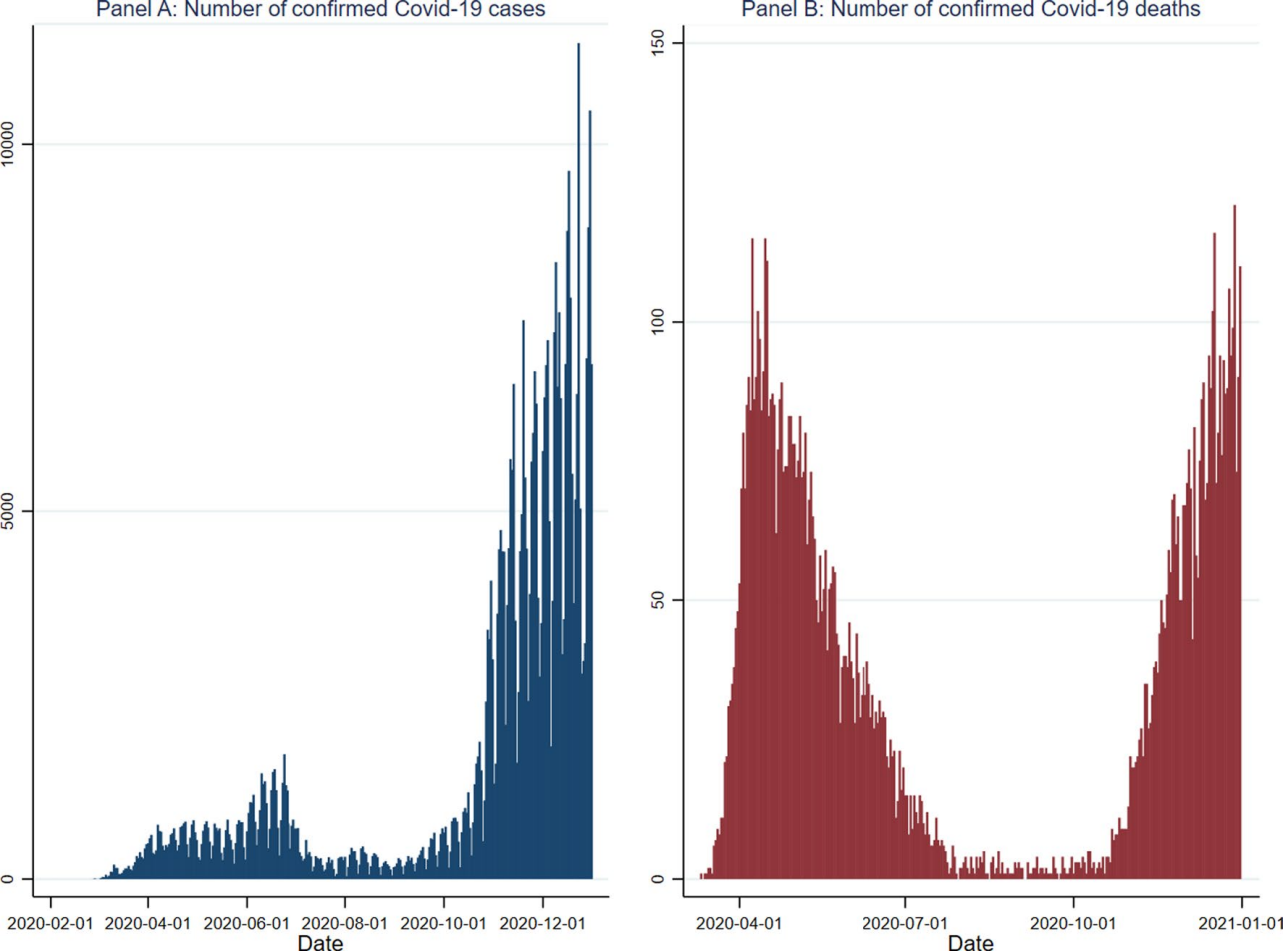
Covid-19 pandemic in Sweden, 2020. Source: Public Health Agency of Sweden; accessed on February 15, 2021

In 2020, Sweden experienced two distinct waves of the Covid-19 pandemic; see Fig. 1. The first wave started in March and lasted until mid-June. In early November of the same year, the number of cases started increasing again and continued into the next year.

The first wave of the pandemic as measured by the number of daily confirmed cases is most likely under-reported due to limited testing at that stage of the pandemic.

#### Overall utilization of primary care

**Fig. 2:**
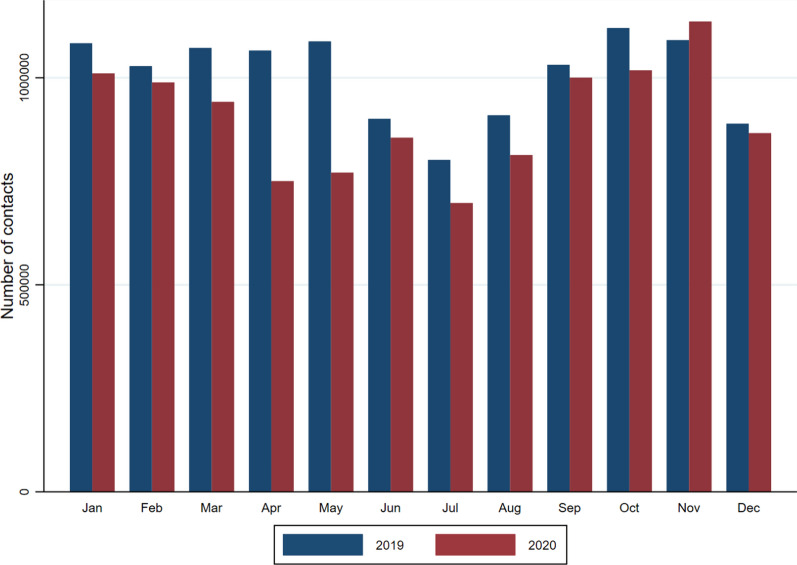
Total primary care consultations, Sweden (selected regions), 2019 and 2020. Source: VTDB 2019 and 2020

Figure 2 shows the total number of patient consultations in 2019 and 2020, respectively, for the current sample of Swedish regions with complete data for both years. Utilization in the two pre-pandemic months of 2020 (January and February) was similar to the same months in the baseline year of 2019. As the first wave of the pandemic grew in size, the number of primary care consultations started to decrease. The reductions in April and May of 2020 were particularly large and appear to coincide with the peak in the number of confirmed deaths. They also contrast with the increase in utilization over February–May that is seen in 2019 and that is partly driven by seasonal respiratory tract infections.

After the large decrease in the second quarter of 2020, the number of consultations rebounded and continued to increase into the third quarter of the year. The subsequent fall in December of 2020 is comparable to the corresponding month of the previous year. In addition to these general findings, there was no immediate covariance between pandemic levels and utilization of primary care services within individual regions.

#### Utilization by type of consultation

Table 1 reports the total number of consultations by type for the regions with complete data in both years (see also Additional file 1: Figure S1).

**Table 1:** Primary care consultations by type and year, selected regions

Consultation type	Year	
	2019 N %	2020 N %
Clinic visits	10,499,364	8,819,487
	86.9	81.3
Telemedicine contacts	320,873	269,609
	2.7	2.5
Home visits	159,844	171,380
	1.3	1.6
Telephone/letter contacts	1,099,187	1,587,442
	9.1	14.6
Total	12,079,268	10,847,918

Source: VTDB 2019 and 2020

In 2019, remote contacts by means of video- and chat-based technologies and telephone/letter contacts made up a combined 12 percent of all consultations. In 2020, this share increased to 17 percent overall. However, the data also show that the actual increase in the number of remote contacts is substantially smaller than the decrease in the number of on-site visits. Furthermore, the changes in remote types of contacts appear to differ as telephone and letter accounted for the largest increase in remote contacts in 2020 whereas video-based consultations actually decreased in the current sample, both in absolute numbers and as a share of total consultations.

While non-physician home visits increased somewhat from 2019 to 2020, these types of visits account for a very small share of total consultations. The reductions in the number of monthly on-site visits for most months of 2020 suggest that the total number of primary care consultations of that year was lower than it should have been in the absence of the pandemic. While changes vary across the regions, it is estimated that the total number of primary care consultations in Sweden fell by on average 11.84 percent in 2020 (from  + 1.3 percent to − 22.13 percent; see Additional file 1: Figure S2). This relative reduction suggests a shortfall of some 2.7 million primary care consultations in 2020 as a result of the Covid-19 pandemic.^2^

#### Utilization by age and sex

With respect to the effect on various age groups, the data show that the reduction in primary care consultations affected those over 65 years of age more compared with the younger patient groups (see Additional file 1: Figure S3). Regarding any changes in primary care utilization of women compared with men, findings suggest that the relative share of men to women remained stable throughout 2020 and also was very similar to the baseline year of 2019 (see Additional file 1: Table S1 for details).

## Discussion

How health systems are able to adjust to unforeseen events, such as the on-going pandemic, is an issue of critical importance for future policy development in most countries. This study finds that even in a country with a relatively well-developed primary care system and, moreover, a comparably low level of primary care utilization rate per capita, a significant number of patients have not received care as expected in the first year of the Covid-19 pandemic. While remote types of services appear to have increased as the pandemic continued to affect service delivery, such types of patient consultations have been unable to make up for the overall reduction in primary care use in the current case.

The findings also suggest that the fall in services has affected both men and women to a similar extent. Given what was known about the SARS-CoV-2 virus during the initial stages of the pandemic, this would be an expected finding. More generally, the relatively larger share of women to men in primary care utilization in Sweden is consistent with that of most other countries [9].

However, the results also indicate that patients over 65 years of age saw a particularly large reduction in primary care utilization. While this would be in line with the general recommendations by disease control agencies to specifically protect this demographic group from avoidable contacts, it may imply that some patients’ health needs have not been fully met during the pandemic. The potential long-term effects that this may have, in particular as the pandemic has continued into 2021, is a matter of both policy and research relevance.

The present study describes the broad changes in the utilization of primary care services in Sweden during the first and second wave of the Covid-19 pandemic. As such, it provides a basis on which to plan further, detailed investigations into the nature of the observed changes. Among other questions, future analyses will assess the extent to which some groups were more affected than others. For example, the effect of the pandemic on patients with chronic conditions, such as hypertension and diabetes, need to be investigated.

Another large group of patients are those seeking mental health care that may also have been particularly hit by the effects of the pandemic [10]. For example, increased suicide rates have been reported in Japan [11], while in another study covering 21 countries, no such increases were reported [12].

A further issue is the type of remote contacts that have been used by providers during the pandemic [13]. In the present study, traditional types of remote patient consultations, such as telephone and letter, saw a larger increase compared with more modern approaches, such as digital telemedicine (video and chat-based). A general policy issue relates to the understanding of the effectiveness of these different types of remote care and how they should be directed across various patient groups [14, 15].

## Limitations

One limitation of the study is the incompleteness of the data for the baseline year of 2019. The database was initiated in 2019 and not all regions reported data to the system from the beginning of the year. Five regions joined later in the year and two regions began reporting data in January of 2020. However, the current sample covers two-thirds of the Swedish regions, which suggests that the study’s findings are largely representative of the country as a whole.

An additional limitation of the data is the absence of a patient identification number. For the purposes of the current study, this limitation was not of immediate concern. However, it does prohibit any analysis of individual patients’ utilization of services across repeated illness episodes, an issue that may be of interest in future studies.

## Supplementary Information

**Additional file 1: Table S1.** Primary care consultations by month and sex, Sweden (selected regions), 2019 and 2020. **Figure S1.** Primary care consultations by type [On-site visits (left-hand side) and Remote contacts (right-hand side)], Sweden (selected regions), 2019 and 2020. **Figure S2.** Estimated relative differences in total consultations between 2020 and 2019, Sweden (selected regions; %). **Figure S3.** Primary care consultations by age groups and Quarter, Sweden (selected regions), 2019 and 2020.

## Data Availability

The dataset supporting the conclusions of this study is available in the OSF repository, https://osf.io/rp6fb/?view_only=6776f80073e940e18937b469f8e84b2e.

## Abbreviations

VTDB: Väntetidsdatabasen (a national primary care database)

## Footnotes

1 The complete sample sizes for the two years before adjusting for missing data in 2019 were 15 million (in 2019) and 23 million (in 2020), suggesting an annual overall utilization rate of around 2.3 primary care consultations per person.

2 11.84 percent on top of the total number of consultations in 2020 of some 23 million.

## References

[1] Levene LS, Seidu S, Greenhalgh T, Khunti K (2020). Pandemic threatens primary care for long term conditions. BMJ.

[2] Stokel-Walker C (2020). Why telemedicine is here to stay. BMJ.

[3] Baum A, Schwartz M (2020). Admissions to Veterans Affairs hospitals for emergency conditions during the Covid-19 pandemic. JAMA.

[4] OECD (2021). Strengthening the frontline: how primary health care helps systems adapt during the Covid-19 pandemic.

[5] Mann DM, Chen J, Chunara R, Testa PA, Nov O (2020). Covid-19 transforms health care through telemedicine: evidence from the field. J Am Med Inform Assoc.

[6] Mehrotra A, Ray K, Brockmeyer DM, Barnett ML, Bender JA (2020). Rapidly converting to “virtual practices”: outpatient care in the era of Covid-19. NEJM Catalyst.

[7] Richardson E, Aissat D, Williams GA, Fahy N (2020). Keeping what works: remote consultations during the Covid-19 pandemic. Eurohealth.

[8] Anell A, Glenngård AH, Merkur S (2012). Sweden—health system review.

[9] OECD (2020). Health at a glance: Europe 2020—state of health in the EU cycle.

[10] Cullen W, Gulati G, Kelly BD (2020). Mental health in the Covid-19 pandemic. QJM Int J Med.

[11] Sakamoto H, Ishikane M, Chaznavi C, Ueda P (2021). Assessment of suicide in Japan during the Covid-19 pandemic vs previous years. JAMA Netw Open.

[12] Pirkis J, John A, Shin S, DelPozo-Banos M, Arya V, Analuisa-Aguilar P (2021). Suicide trends in the early months of the Covid-19 pandemic: an interrupted time-series analysis of preliminary data from 21 countries. Lancet Psychiatry.

[13] Patel SY, Rose S, Barnett ML, Huskamp HA, Uscher-Pines L, Mehrotra A (2021). Community factors associated with telemedicine use during the Covid-19 pandemic. JAMA Network Open.

[14] Ekman B, Thulesius H, Wilkens J, Lindgren A, Cronberg O, Arvidsson E (2019). Utilization of digital primary care in Sweden: descriptive analysis of claims data on demographics, socioeconomics, and diagnoses. Int J Med Inform.

[15] Hollander JE, Carr BG (2020). Virtual perfect? Telemedicine for Covid-19. N Engl J Med.

