# Supplementary material for: Impact of the Covid-19 pandemic on primary care utilization: evidence from Sweden using national register data
Source: BMC Res Notes. 2021 Nov 24;14:424. doi: 10.1186/s13104-021-05839-7 (PMC8611625; doi:10.1186/s13104-021-05839-7)
Supplement: Supplementary file 1 — Additional file 1: Table S1. Primary care consultations by month and sex, Sweden (selected regions), 2019 and 2020. Figure S1. Primary care consultations by type [On-site visits (left-hand side) and Remote contacts (right-hand side)], Sweden (selected regions), 2019 and 2020. Figure S2. Estimated relative differences in total consultations between 2020 and 2019, Sweden (selected regions; %). Figure S3. Primary care consultations by age groups and Quarter, Sweden (selected regions), 2019 and 2020. [file 13104_2021_5839_MOESM1_ESM.docx]

Ekman et al. (2021) Primary care and Covid-19.

**Supplementary material**

**Table S.1 Primary care consultations by month and sex, Sweden (selected regions), 2019 and 2020**

|  |  | **2019** |  |  |  | **2020** |  |  |
| --- | --- | --- | --- | --- | --- | --- | --- | --- |
| **Month** | **Female** | **Male** | **Total** | **Male share (%)** | **Female** | **Male** | **Total** | **Male share (%)** |
| January | 637,986 | 445,191 | 1,083,177 | 41 | 595,955 | 414,503 | 1,010,458 | 41 |
| February | 605,089 | 422,881 | 1,027,970 | 41 | 583,193 | 405,589 | 988,782 | 41 |
| March | 631,071 | 441,137 | 1,072,208 | 41 | 551,828 | 389,799 | 941,627 | 41 |
| April | 625,018 | 440,546 | 1,065,564 | 41 | 439,530 | 310,718 | 750,248 | 41 |
| May | 639,253 | 448,436 | 1,087,689 | 41 | 452,290 | 318,620 | 770,910 | 41 |
| June | 525,586 | 375,186 | 900,772 | 42 | 498,549 | 356,511 | 855,060 | 42 |
| July | 462,098 | 339,558 | 801,656 | 42 | 404,046 | 293,232 | 697,278 | 42 |
| August | 527,652 | 381,578 | 909,230 | 42 | 477,537 | 335,800 | 813,337 | 41 |
| September | 608,812 | 422,518 | 1,031,330 | 41 | 594,756 | 405,417 | 1,000,173 | 41 |
| October | 664,491 | 455,471 | 1,119,962 | 41 | 604,771 | 413,371 | 1,018,142 | 41 |
| November | 638,887 | 451,858 | 1,090,745 | 41 | 667,767 | 467,845 | 1,135,612 | 41 |
| December | 514,431 | 374,534 | 888,965 | 42 | 510,172 | 356,119 | 866,291 | 41 |
| **Total** | **7,080,374** | **4,998,894** | **12,079,268** |  | **6,380,394** | **4,467,524** | **10,847,918** |  |

Source: VTDB 2019 and 2020.

**Figure S.1 Primary care consultations by type (On-site visits (left-hand side) and Remote contacts (right-hand side)), Sweden (selected regions), 2019 and 2020**


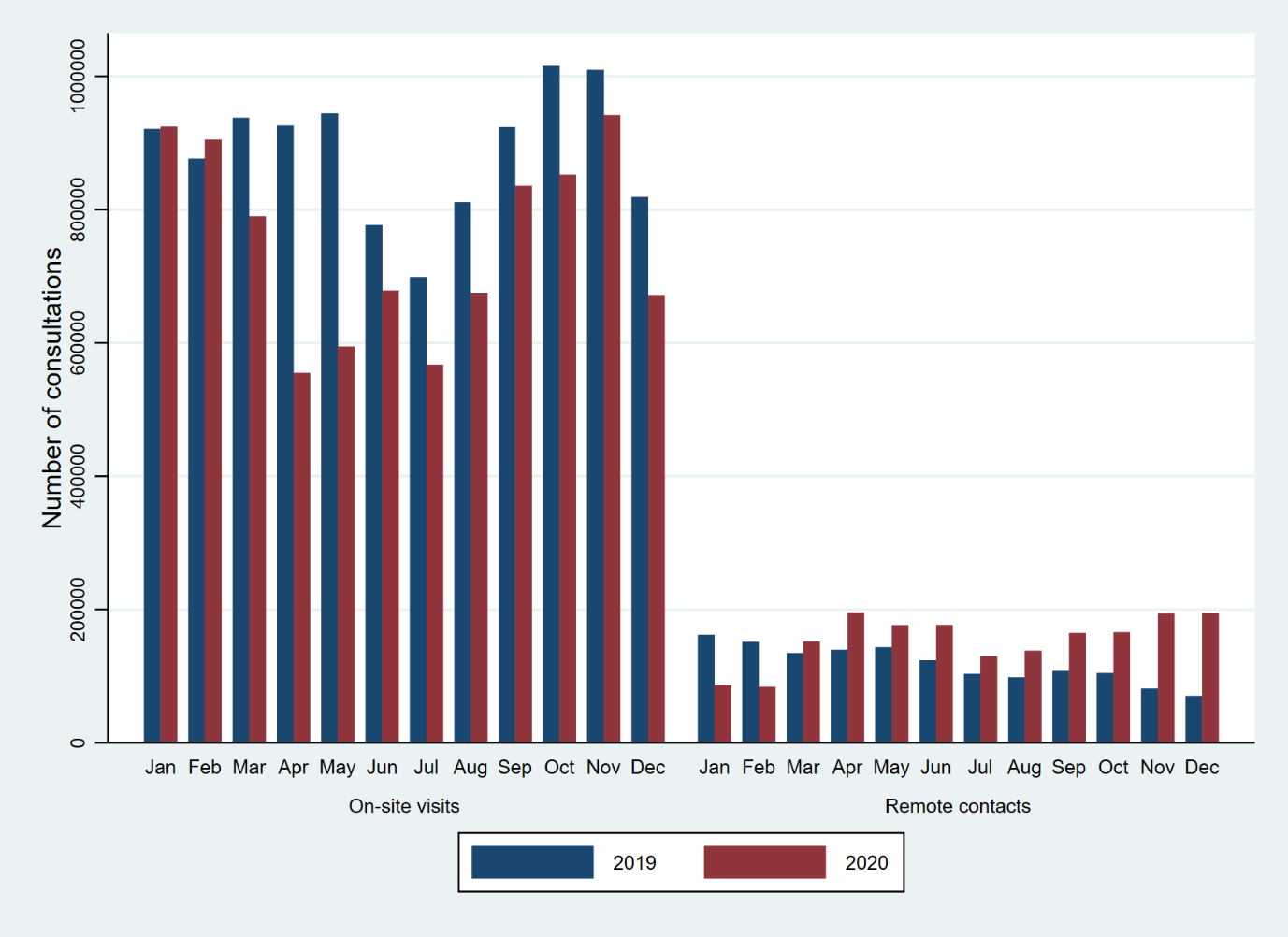


Source: VTDB 2019 and 2020.

**Figure S.2 Estimated relative differences in total consultations between 2020 and 2019, Sweden (selected regions; %)**

Source: VTDB 2019 and 2020.

**Figure S.3 Primary care consultations by age groups and Quarter, Sweden (selected regions), 2019 and 2020**


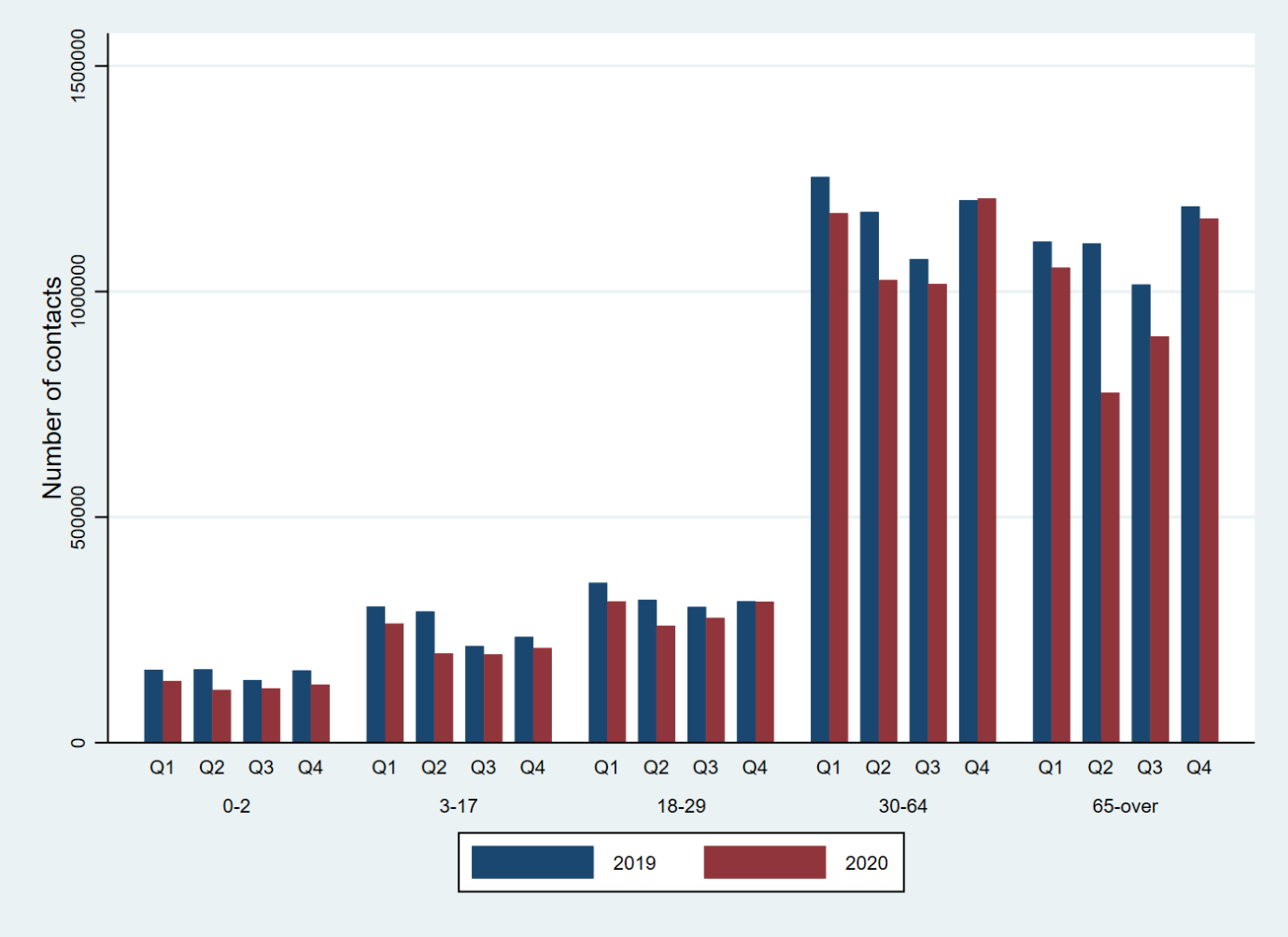


Source: VTDB 2019 and 2020.
